# Usefulness of FT3 to FT4 Ratio to Predict Mortality in Euthyroid Patients With Prior Cardiovascular Events Undergoing PCI: Five-Year Findings From a Large Single-Center Cohort Study

**DOI:** 10.3389/fendo.2021.700349

**Published:** 2021-07-05

**Authors:** Deshan Yuan, Sida Jia, Pei Zhu, Ce Zhang, Yue Liu, Ru Liu, Jingjing Xu, Xiaofang Tang, Xueyan Zhao, Runlin Gao, Yuejin Yang, Bo Xu, Zhan Gao, Jinqing Yuan

**Affiliations:** Fu Wai Hospital, National Center for Cardiovascular Diseases, Peking Union Medical College & Chinese Academy of Medical Sciences, Beijing, China

**Keywords:** FT3/FT4 ratio, thyroid hormones, coronary artery disease, percutaneous coronary intervention, prognosis

## Abstract

**Background:**

In euthyroid patients undergoing percutaneous coronary intervention (PCI), it is still unclear whether free triiodothyronine to free thyroxine (FT3/FT4) ratio can predict the recurrence of cardiovascular events (CVEs). We aim to investigate its association with recurrent long-term adverse events in this population.

**Methods:**

3549 euthyroid patients with prior CVEs history undergoing PCI were consecutively enrolled in our study and subsequently divided into three FT3/FT4 ratio tertiles (T1<2.41, n=1170; 2.41≤T2<2.75, n=1198; T3>2.75, n=1181). The primary endpoint was major adverse cardiovascular and cerebrovascular event (MACCE), a composite of all-cause death, myocardial infarction, stroke and revascularization. The secondary endpoints were all-cause death and cardiac death.

**Results:**

The median follow-up time was 5 years. The incidence of all-cause death, cardiac death and MACCE were significantly higher among patients in the lowest FT3/FT4 tertile (P<0.05). After adjustment of confounding factors, decreased FT3/FT4 ratio was independently associated with an increased risk of all-cause death (HR 1.82, 95% CI 1.13-2.93, P=0.014), cardiac death (HR 1.90, 95% CI 1.04-3.46, P=0.036) and MACCE (HR 1.33, 95% CI 1.10-1.60, P=0.003) which was driven mainly by all-cause death.

**Conclusions:**

In euthyroid patients with prior cardiovascular events undergoing PCI, FT3/FT4 ratio might be a potential predictor of all-cause and cardiac mortality. Routine assessment of FT3/FT4 ratio might be a simple and effective tool for risk stratification in this specific patient population.

## Introduction

Despite effective secondary prevention such as lifestyle management and optimal drug treatment, patients with coronary artery disease (CAD) who survived prior cardiovascular events (CVEs) remain at high-risk for recurrences (1). Consequently, identifying potential risk parameters are essential for both early risk stratification and future prognostication in CAD patients with prior CVEs. Thyroid hormones exert a wide range of effects on the heart and cardiovascular system (2, 3). Overt and subclinical thyroid dysfunction (4–6), as well as low T3 syndrome (7), can increase CAD risk. Recent observational data suggested that variation in thyroid hormones within reference range also deteriorates outcomes in specific clinical conditions, possibly through impairment of peripheral thyroxin deiodination and downregulation of deiodinase activity (8–11). Moreover, we have previously found that the reduction of FT3/FT4 ratio, a surrogate marker of thyroxin deiodination, increased the risk of long-term adverse CVEs in euthyroid patients with three-vessel CAD (12). To the best of our knowledge, the potential relationship of FT3/FT4 ratio and recurrent adverse outcomes in euthyroid patients with prior CVEs has not been investigated, especially in patients treated with percutaneous coronary intervention (PCI). Therefore, we aim to determine whether FT3/FT4 ratio is capable of predicting long-term CVEs recurrence in euthyroid patients undergoing PCI, through analysis from a real-world, prospective, observational cohort of Chinese patients.

## Materials and Methods

### Study Participants

This prospective, observational cohort study consecutively enrolled 10724 patients with CAD hospitalized from January 2013 to December 2013 to receive PCI in Fu Wai Hospital. The study protocol was approved by the Institutional Ethic Committee of Fu Wai Hospital and complied with the Declaration of Helsinki. All enrolled patients provided written informed consent before participating in the study. The exclusion criteria include: 1) patient without detailed data of thyroid function tests (n=268); 2) patient with prior/current thyroid disorders and/or on medications that might affect thyroid function test including amiodarone, lithium, glucocorticoids, thyroid hormones, sex hormones, phenytoin sodium (n=1948); 3) patient without history of prior CVEs [defined as myocardial infarction (MI), PCI, coronary artery bypass grafting (CABG), stroke or peripheral arterial disease before admission] (n=4959). Fasting blood samples were drawn from all patients within 24 hours after admission. Thyroid function profile was measured by direct chemiluminescence method (ADVIA Centaur, Siemens, USA). The reference intervals were as follows: FT3, 2.3-4.2 pg/ml; FT4, 0.89-1.76 ng/dl; thyroid stimulating hormone (TSH), 0.55-4.78 µIU/mL; total triiodothyronine (TT3), 0.60-1.81 ng/ml; total thyroxine (TT4), 4.5-10.9 µg/dl. Patients were considered euthyroid if the serum TSH, FT3, FT4 falls within the reference range. Additionally, structure parameters of thyroid homeostasis including secretory capacity of the thyroid gland (SPINA-GT) and sum activity of peripheral deiodinases (SPINA-GD) were also calculated (13). SPINA-GT=[β_T_×(D_T_+TSH)×TT4]/(α_T_×TSH)(reference range: 1.4-8.7 pmol/s). SPINA-GD=[β_31_×(K_M1_+FT4)×TT3]/(α_31_×FT4)(reference range: 20-60 nmol/s). Constants in the equations were as follows: β_T_ = 1.1×10^-6^/s, D_T_=2.75mIU/L,α_T_=0.1/L, β_31 =_ 8×10^-6^/s, K_M1 =_ 500 nmol/L, and α_31 =_ 0.026/L.

### Procedure and Medications

Before the procedure, patients receiving selective PCI were treated with aspirin (300mg) and ticagrelor (loading dose, 180mg) or clopidogrel (loading dose, 300mg), except for patients already on dual antiplatelet therapy; for patients with ACS receiving emergency PCI, the same dose of aspirin and ticagrelor or clopidogrel (loading dose, 300-600 mg) were administered as soon as possible. All patients were administered with unfractionated heparin (100 U/kg), and interventional cardiologist decided whether to use glycoprotein IIb/IIIa antagonist according to the clinical conditions and coronary lesions during the procedure. After the procedure, the dual antiplatelet therapy including aspirin (100mg daily), ticagrelor (90mg, twice daily) or clopidogrel (75mg, daily) were recommended for at least 1 year. The choice of equipment and techniques during PCI was at the discretion of the physicians.

### Follow-Up and Outcomes

Regular follow-up assessment of patients was performed at five time points (1-month, 6-month, 12-month, 2-year, and 5-year after the discharge) through outpatient clinical visits or telephone interview. The primary endpoint was major adverse cardiovascular and cerebrovascular events (MACCE), defined as the occurrence of target lesion revascularization, MI, stroke and all-cause death during the follow-up. The secondary outcomes included all-cause death and cardiac death. Myocardial infarction (MI) was defined according to the third universal definition of myocardial infarction (14). Target lesion revascularization was defined as unplanned revascularization (PCI or CABG) for angina or ischemia related to the target lesion. Cardiac death was defined as any death without a clear non-cardiac cause.

### Definition of Clinical Status

Diabetes was defined as newly diagnosed diabetes (HbA1c≥6.5%, FPG≥7.0mmol/L, oral glucose tolerance test≥11.1 mmol/L), or known diabetes and current use of hypoglycemic drugs or insulin. Hypertension was defined as systolic blood pressure≥140 mmHg and/or diastolic blood pressure≥90 mmHg and/or current use of antihypertensive drugs. Low-density lipoprotein cholesterol ≥3.4mmol/L, fasting total cholesterol ≥5.2 mmol/L, triglyceride≥1.7 mmol/L, high-density lipoprotein cholesterol <1.0 mmol/L and/or chronic use of lipid-lowering drugs were considered criteria for dyslipidemia. Left main disease was defined as stenosis of ≥50% in left main coronary artery, and three-vessel disease was defined as stenosis of ≥50% in all three main coronary arteries (right coronary artery, left circumflex artery and left anterior descending artery) confirmed by coronary angiography.

### Statistical Analysis

Clinical and laboratory characteristics of patients were tabulated according to tertiles of FT3/FT4 ratio. Continuous variables were presented as mean and standard deviation, while categorical variables were presented as frequency and percentage. Differences of continuous and categorical variables were analyzed by analysis of Variance or Kruskal-Wallis test and χ^2^ test or Fisher’s exact test, as appropriate. Event-free survival rates were presented by Kaplan-Meier curves and compared by log-rank test. The association between FT3/FT4 ratio and clinical outcomes were analyzed by Cox proportional hazards regression. In the multivariable analysis, the following variables were chosen because of their statistical significance in the univariable analysis or clinical importance: age, sex, BMI, diabetes, hypertension, dyslipidemia, family history of CAD, smoking, clinical presentation [stable angina pectoris or acute coronary syndrome (ACS)], LVEF, TSH, T3, T4, HbA1c, LDL-C, hs-CRP, eGFR, lesion vessels, Left main/three vessel disease, SYNTAX score, complete revascularization, Number of stents and DES implantation. The statistical analyses were performed with SPSS version 25.0 software (IBM Corporation, Chicago, IL) and P*<* 0.05 was considered statistically significant.

## Results

### Baseline Characteristics

A total of 3,549 patients eligible were ultimately included in the study and subsequently divided into three groups according to the tertiles of FT3/FT4 ratio (T1<2.41, n=1170; 2.41≤T2<2.75, n=1198; T3≥2.75, n=1181). Baseline clinical and laboratory characteristics of the study patients are presented in **Table 1**. Patients in the lowest tertile of FT3/FT4 ratio were much older and the proportion of male was smaller than in the other two groups (all *P*<0.05). Significant differences were also observed in terms of other baseline characteristics according to tertiles of FT3/FT4 ratio. In general, the reduction of FT3/FT4 ratio was associated with worse cardiovascular risk profile. Specifically, patients with lower levels of FT3/FT4 ratio showed a larger burden of concomitant diseases such as diabetes and hypertension (all *P*<0.05). They were also prone to ACS, as well as left main or three-vessel CAD (all *P*<0.05). Moreover, these patients were more likely to have higher BMI, higher HbA1c, higher LDL cholesterol, higher hs-CRP, lower LVEF, lower estimated GFR and lower rate of complete revascularization (all *P*<0.05). In terms of thyroid homeostasis parameters, SPINA-GD was significantly lower while SPINA-GT was significantly higher in the lowest FT3/FT4 ratio group (all *P*<0.05).

**Table 1:** Baseline clinical and laboratory characteristics of the study patients according to the FT3/FT4 ratio tertiles.

Variables	T1 (n=1170)	T2 (n=1198)	T3 (n=1181)	*P* value
**Age, years**	60.6 ± 10.3	58.8 ± 10.0	57.2 ± 9.9	<0.001
**Male**	875 (74.8)	1006 (84.0)	1049 (88.8)	<0.001
**BMI, kg/m^2^**	25.9 ± 3.2	26.1 ± 3.0	26.2 ± 3.0	0.048
**Diabetes**	673 (57.5)	572 (47.7)	501 (42.4)	<0.001
**Hypertension**	840 (71.8)	795 (66.4)	745 (63.1)	<0.001
**Dyslipidemia**	858 (73.3)	850 (71.0)	841 (71.2)	0.370
**Family history of CAD**	291 (24.9)	312 (26.1)	300 (25.4)	0.807
**Smoking**	692 (59.1)	766 (63.9)	793 (67.1)	<0.001
**Clinical presentation**				<0.001
**SAP**	534 (45.6)	605 (50.5)	664 (56.2)	
**ACS**	636 (54.4)	593 (49.5)	517 (43.8)	
**LVEF, %**	60.7 ± 8.6	61.9 ± 7.5	62.1 ± 7.4	<0.001
**Laboratory findings**				
** TSH, µ IU/mL**	1.89 ± 0.94	1.86 ± 0.92	1.83 ± 0.91	0.352
** T3, ng/mL**	1.01 ± 0.20	1.07 ± 0.21	1.12 ± 0.22	<0.001
** T4, μg/dL**	9.18 ± 1.79	8.63 ± 1.69	8.13 ± 1.55	<0.001
** FT4, ng/dL**	1.28 ± 0.14	1.15 ± 0.11	1.04 ± 0.09	<0.001
** FT3, pg/mL**	2.78 ± 0.27	2.96 ± 0.28	3.15 ± 0.28	<0.001
** FT3/FT4**	2.18 ± 0.18	2.58 ± 1.00	3.02 ± 0.22	<0.001
** SPINA-GD, nmol/s**	15.14 ± 3.05	17.67 ± 3.40	20.39 ± 4.10	<0.001
** SPINA-GT, pmol/s**	3.72 ± 1.45	3.53 ± 1.39	3.35 ± 1.29	<0.001
**FBG, mmol/L**	6.7 ± 2.5	6.1 ± 1.9	5.9 ± 1.6	<0.001
** HbA1c, %**	7.0 ± 1.4	6.6 ± 1.2	6.5 ± 1.1	<0.001
**TC, mmol/L**	4.2 ± 1.0	4.0 ± 1.0	4.0 ± 1.0	<0.001
**TG, mmol/L**	1.8 ± 1.2	1.7 ± 1.0	1.7 ± 1.0	0.002
**LDL-C, mmol/L**	2.4 ± 0.9	2.4 ± 0.9	2.3 ± 0.9	0.020
**HDL-C, mmol/L**	1.1 ± 0.3	1.0 ± 0.3	1.0 ± 0.2	<0.001
** hs-CRP, mg/L**	3.5 ± 4.0	2.7 ± 3.2	2.1 ± 2.5	<0.001
**eGFR, ml/min**	87.5 ± 16.2	91.3 ± 14.8	94.1 ± 13.3	<0.001
**Lesion vessels**	1.4 ± 0.6	1.4 ± 0.7	1.4 ± 0.6	0.147
**LM/three-vessel disease, n%**	578 (49.4)	582 (48.6)	517 (43.8)	0.013
**SYNTAX score**^**a**^	11.6 ± 8.7	10.8 ± 8.2	11.1 ± 8.0	0.053
**Complete revascularization**	487 (41.6)	543 (45.3)	550 (46.6)	0.043
**Number of stents**	1.7 ± 1.1	1.8 ± 1.1	1.7 ± 1.1	0.787
**DES implantation, n%**	1066 (91.1)	1104 (92.2)	1092 (92.5)	0.452
**Medications at discharge**				
**Aspirin**	1153 (98.5)	1180 (98.5)	1170 (99.1)	0.396
**Clopidogrel**	1155 (98.7)	1173 (97.9)	1164 (98.6)	0.255
**β-blocker**	1072 (91.6)	1109 (92.6)	1076 (91.1)	0.420
**ACEI/ARB**	669 (57.2)	668 (55.8)	669 (56.6)	0.780
**Statin**	1103 (94.3)	1148 (95.8)	1132 (95.9)	0.116
**CCB**	584 (49.9)	560 (46.7)	552 (46.7)	0.206
**Nitrate**	1137 (97.2)	1172 (97.8)	1153 (97.6)	0.579

Values are presented as mean ± standard deviation or number (%).

ACEI, angiotensin-converting enzyme inhibitors; ACS, acute coronary syndrome; ARB, angiotensin II receptor blockers; BMI, body mass index; CAD, coronary artery disease; CCB, calcium channel blocker; DES, drug-eluting stent; eGFR, estimated glomerular filtration rate; FT3, free triiodothyronine; FT4, free thyroxine; FBG, fasting blood glucose; hs-CRP, high sensitivity C-reactive protein; HbA1c, Hemoglobin A1c; HDL-C, high-density lipoprotein cholesterol; LDL-C, low-density lipoprotein cholesterol; LM, left main disease; LVEF, left ventricular ejection fraction; SAP, stable angina pectoris; SINA-GD, sum activity of deiodinases, SPINA-GT, thyorid’s secretory capacity, TSH, thyroid-stimulating hormone; T3, total triiodothyronine; T4, total thyroxine; TC, total cholesterol; TG, triglycerides.

a Calculated using an online calculator (http://www.syntaxscore.com/) by a dedicated research group blinded to the clinical data.

### Relation of Risk Factors and Recurrent Adverse Events

**Figure 1:**
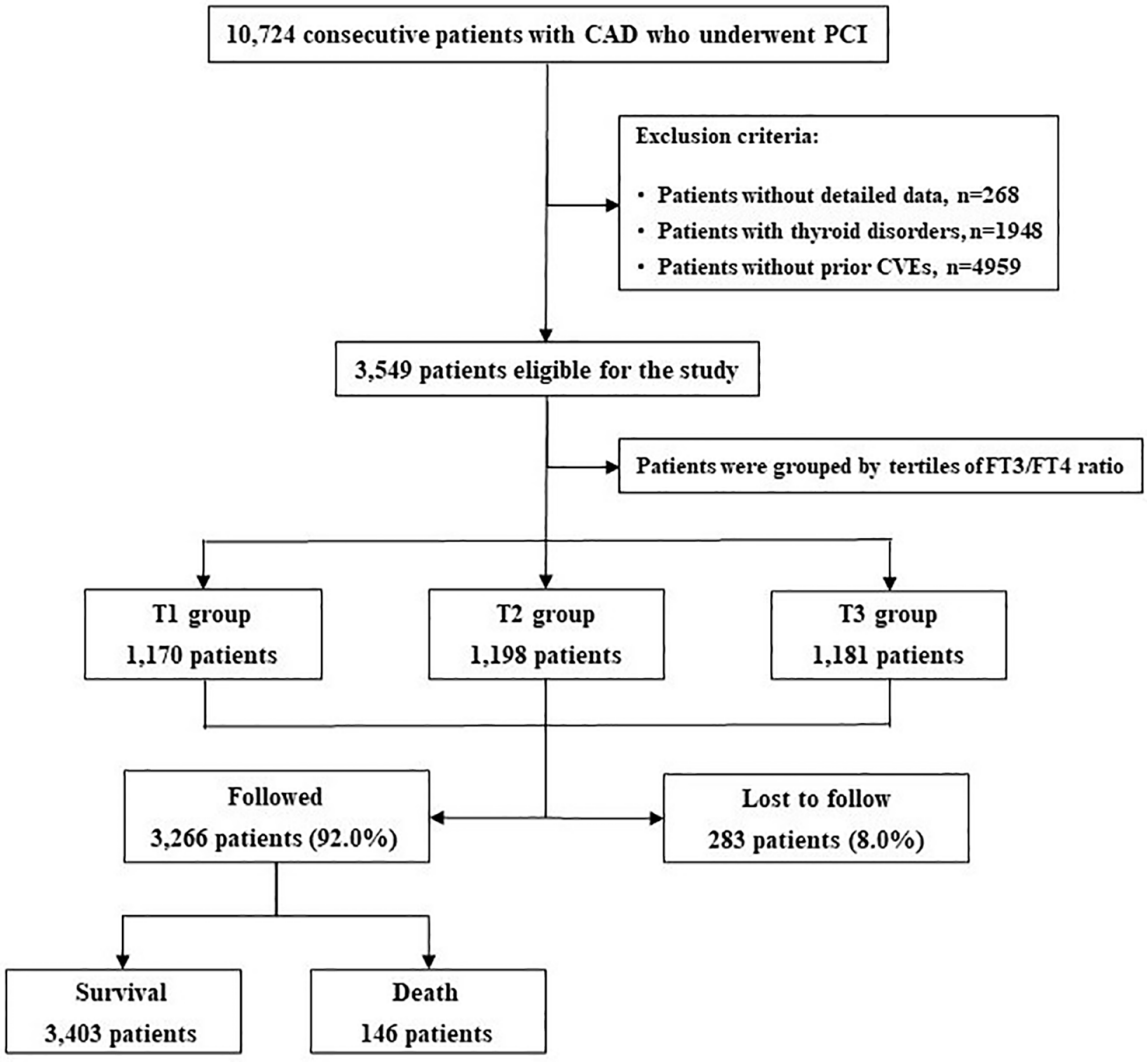
Patient Flowchart. CAD, Coronary Artery Disease; PCI, CVEs, Cardiovascular events; FT3, Free Triiodothyronine; FT4, Free Thyronine; Percutaneous Coronary Intervention.

The overall median follow-up time was 5.0 years (interquartile range 3.0-5.1 years), and the response rate was 92% (**Figure 1**). During this period, 855 (24.1%) and 146 (4.1%) patients experienced MACCE and all-cause death, respectively. Patients in the MACCE group had a higher incidence of diabetes, hypertension, left main and/or three-vessel disease, with higher levels of FBG, HbA1c, hs-CRP and reduced LVEF (all *P*<0.05). With regard to the PCI procedure, patients suffering MACCE had higher SYNTAX score, lower rate of complete revascularization and DES use (all *P*<0.05). Of note, the FT3/FT4 ratio was also statistically lower in patients with MACCE (*P*=0.001) (**Table 2**).

**Table 2:** Baseline clinical and laboratory characteristics of the study patients with and without MACCE at 5 years.

Variables	Without MACCE (n=2694)	With MACCE (n=855)	*P* value
**Age, years**	58.7 ± 10.1	59.3 ± 10.2	0.138
**Male**	2213 (82.1)	717 (83.9)	0.250
**BMI, kg/m^2^**	26.0 ± 3.0	26.1 ± 3.2	0.713
**Diabetes**	1300 (48.3)	446 (52.2)	0.046
**Hypertension**	1782 (66.1)	598 (69.9)	0.040
**Dyslipidemia**	1921 (71.3)	628 (73.5)	0.225
**Family history of CAD**	682 (25.3)	221 (25.9)	0.751
**Smoking**	1701 (63.1)	550 (64.3)	0.530
**Clinical presentation**			0.259
**SAP**	1311 (48.7)	435 (50.9)	
**ACS**	1383 (51.3)	420 (49.1)	
**LVEF, %**	61.7 ± 7.8	61.1 ± 8.2	0.044
**Laboratory findings**			
** TSH, µ IU/mL**	1.85 ± 0.92	1.88 ± 0.93	0.384
** T3, ng/mL**	1.07 ± 0.21	1.07 ± 0.24	0.376
** T4, μg/dL**	8.65 ± 1.73	8.63 ± 1.74	0.816
** FT4, ng/dL**	1.16 ± 0.15	1.17 ± 0.16	0.141
** FT3, pg/mL**	2.97 ± 0.32	2.94 ± 0.30	0.002
** FT3/FT4**	2.61 ± 0.38	2.56 ± 0.39	0.001
** SPINA-GD, nmol/s**	17.81 ± 4.12	17.00 ± 4.61	0.022
** SPINA-GT, pmol/s**	3.52 ± 1.38	3.44 ± 1.34	0.455
**FBG, mmol/L**	6.2 ± 2.0	6.4 ± 2.2	0.024
**HbA1c, %**	6.7 ± 1.2	6.8 ± 1.3	0.012
**TC, mmol/L**	4.1 ± 1.1	4.1 ± 1.0	0.846
**TG, mmol/L**	1.7 ± 1.1	1.8 ± 1.0	0.645
**LDL-C, mmol/L**	2.4 ± 0.9	2.4 ± 0.9	0.650
**HDL-C, mmol/L**	1.0 ± 0.3	1.0 ± 0.3	0.362
** hs-CRP, mg/L**	2.7 ± 3.2	3.0 ± 3.6	0.009
**eGFR, ml/min**	91.2 ± 14.9	90.3 ± 15.4	0.112
**Lesion vessels**	1.4 ± 0.6	1.4 ± 0.6	0.389
**LM/three-vessel disease, n%**	1216 (45.1)	461 (53.9)	<0.001
**SYNTAX score^a^**	11.0 ± 8.1	11.7 ± 9.1	0.034
**Complete revascularization**	1250 (46.4)	330 (38.6)	<0.001
**Number of stents**	1.7 ± 1.1	1.7 ± 1.1	0.330
**DES implantation, n%**	2501 (92.8)	761 (89.0)	<0.001
**Medications at discharge**			
**Aspirin**	2660 (98.7)	843 (98.6)	0.750
**Clopidogrel**	2653 (98.5)	839 (98.1)	0.479
**β-blocker**	2468 (91.6)	789 (92.3)	0.535
**ACEI/ARB**	1531 (56.8)	475 (55.6)	0.513
**Statin**	2570 (95.4)	813 (95.1)	0.709
**CCB**	1287 (47.8)	409 (47.8)	0.974
**Nitrate**	2627 (97.5)	835 (97.7)	0.808

Values are presented as mean ± standard deviation or number (%).

ACEI, angiotensin-converting enzyme inhibitors; ACS, acute coronary syndrome; ARB, angiotensin II receptor blockers; BMI, body mass index; CAD, coronary artery disease; CCB, calcium channel blocker; DES, drug-eluting stent; eGFR, estimated glomerular filtration rate; FT3, free triiodothyronine; FT4, free thyroxine; FBG, fasting blood glucose; hs-CRP, high sensitivity C-reactive protein; HbA1c, Hemoglobin A1c; HDL-C, high-density lipoprotein cholesterol; LDL-C, low-density lipoprotein cholesterol; LM, left main disease; LVEF, left ventricular ejection fraction; SAP, stable angina pectoris; SPINA-GD, sum activity of deiodinases, SPINA-GT, thyroid’s secretory capacity, TSH, thyroid-stimulating hormone; T3, total triiodothyronine; T4, total thyroxine; TC, total cholesterol; TG, triglycerides.

^a^Calculated using an online calculator (http://www.syntaxscore.com/) by a dedicated research group blinded to the clinical data.

### FT3/FT4 Ratio and Recurrent Adverse Events

**Figure 2:**
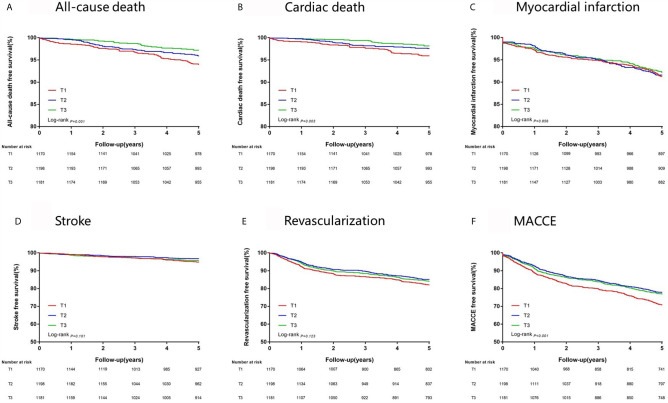
Kaplan-Meier survival analysis on 5-year clinical endpoints among groups. **(A)** All-cause death; **(B)** Cardiac Death; **(C)** Myocardial Infarction; **(D)** Stroke; **(E)** Revascularization; **(F)** MACCE, Major Adverse Cardiac and Cerebrovascular Event.

At 5 years, the incidence of all-cause death, cardiac death and MACCE significantly rises from the highest to the lowest tertile of FT3/FT4 ratio (all *P* < 0.05, **Table 3**). Cumulative free survival of the whole cohort according to FT3/FT4 ratio tertiles was estimated by Kaplan-Meier curves and showed similar results (**Figure 2** **)**.

**Table 3:** Incidence of the primary and secondary outcomes at 5 years.

Events n (%)	T1	T2	T3	P Value
	(n=1091)	(n=1100)	(n=1075)	
**All-cause Death**	67 (6.1)	47 (4.3)	32 (3.0)	**0.002**
**Cardiac Death**	45 (4.1)	28 (2.5)	20 (1.9)	**0.005**
**MACCE**	332 (30.4)	258 (23.5)	265 (24.7)	**<0.001**
**Myocardial infarction**	96 (8.8)	97 (8.8)	90 (8.4)	0.917
**Stroke**	56 (5.1)	37 (3.4)	49 (4.6)	0.117
**Revascularization**	201 (18.4)	172 (15.6)	181 (16.8)	0.219

The bold values represent P<0.05.

Univariable Cox analysis was performed and showed that FT3/FT4 ratio presented strongly negative association with risk of 5-year all-cause death (crude HR 2.12, 95% CI 1.39-3.23, P<0.001), cardiac death (crude HR 2.28, 95% CI 1.35-3.87, P=0.002) and MACCE (crude HR 1.29, 95% CI 1.10-1.52, P=0.002). After adjustment for covariates, FT3/FT4 ratio remained an independent risk factor for 5-year all-cause death (adjusted HR 1.82, 95% CI 1.13-2.93, *P*=0.014), cardiac death (adjusted HR 1.90, 95% CI 1.04-3.46, *P*=0.036) and MACCE (adjusted HR 1.33, 95% CI 1.10-1.60, *P*=0.003). Notably, the significantly higher risk of MACCE in the lowest tertile of FT3/FT4 ratio was mainly driven by all-cause death, because there were no significant differences among groups in the risk for adjusted MI, stroke and revascularization (all *P*>0.05) (**Table 4**). Similarly, the multivariable analysis on 2-year clinical outcomes showed that the decrease of FT3/FT4 ratio was associated with an increased risk of all-cause death (adjusted HR 2.62, 95% CI 1.11-6.21, P=0.028), cardiac death (adjusted HR 3.34, 95% CI 1.04-10.69, P=0.042), and there was a trend towards increased risk of MACCE (adjusted HR 1.27, 95% CI 0.99-1.63, P=0.064) (**Table S1**). Further subgroup analysis showed that there was no potential interaction between FT3/FT4 ratio and sex, age and other common covariates (**Table S2**).

**Table 4:** Univariable and multivariable analysis of the association between FT3/FT4 ratio and endpoints at 5 years.

Outcomes	Crude HR (95% CI)	Crude P-value	Adjusted HR (95% CI)	Adjusted P-value
**All-cause death**				
**T1**	2.12 (1.39‒3.23)	**<0.001**	1.82 (1.13‒2.93)	**0.014**
**T2**	1.45 (0.93‒2.27)	0.105	1.39 (0.87‒2.22)	0.170
**T3**	Reference	‒	Reference	‒
**Cardiac death**				
**T1**	2.28 (1.35‒3.87)	**0.002**	1.90 (1.04‒3.46)	**0.036**
**T2**	1.38 (0.78‒2.45)	0.269	1.31 (0.72‒2.40)	0.374
**T3**	Reference	‒	Reference	‒
**MACCE**				
**T1**	1.29 (1.10‒1.52)	**0.002**	1.33 (1.10‒1.60)	**0.003**
**T2**	0.95 (0.80‒1.12)	0.530	0.96 (0.80‒1.15)	0.635
**T3**	Reference	‒	Reference	‒
**MI**				
**T1**	1.08 (0.81‒1.44)	0.596	1.34 (0.97‒1.86)	0.078
**T2**	1.06 (0.80‒1.42)	0.672	1.14 (0.85‒1.54)	0.385
**T3**	Reference	‒	Reference	‒
**Stroke**				
**T1**	1.16 (0.79‒1.70)	0.445	1.12 (0.71‒1.75)	0.630
**T2**	0.74 (0.48‒1.14)	0.169	0.76 (0.49‒1.20)	0.243
**T3**	Reference	‒	Reference	‒
**Revascularization**				
**T1**	1.15 (0.94‒1.40)	0.186	1.25 (0.99‒1.57)	0.060
**T2**	0.93 (0.76‒1.15)	0.500	0.95 (0.76‒1.18)	0.626
**T3**	Reference	‒	Reference	‒

Model adjusted for age, sex, BMI, diabetes, hypertension, dyslipidemia, family history of CAD, smoking, clinical presentation (stable angina pectoris or ACS), LVEF, TSH, T3, T4, HbA1c, LDL-C, hs-CRP, eGFR, lesion vessels, left main/three vessel disease, SYNTAX score, complete revascularization, number of stents, DES implantation.

CI, confidence interval; HR, hazard ratio; MACCE, major adverse cardiac and cerebrovascular events; MI, myocardial infarction.

## Discussion

In this study, a significant association was noticed between the reduction of FT3/FT4 ratio and an increased risk of all-cause and cardiac mortality in euthyroid CAD patients undergoing PCI. And this association with adverse outcomes remained independent after adjustment for age, sex and other potential confounding risk factors. To our knowledge, the present study is the first one to investigate the prognostic value of FT3/FT4 ratio in euthyroid patients with established CVEs after PCI, and suggested that FT3/FT4 ratio might be a valuable marker for risk stratification and prognostic assessment in this specific patient population.

CAD is a common atherosclerotic cardiovascular disease and brings heavy health and socioeconomic burden both in developed and developing regions worldwide. To make things worse, some CAD related risk factors, notably dyslipidemia and diabetes, are increasingly prevalent and exacerbate this unfavorable situation (15, 16). Clinical practice Guidelines have proposed a range of prevention strategies, either at the general population or individual level, in order to reduce the adverse impact of CAD and related disabilities (1). However, for those patients who have suffered a first CVE, the risk for subsequent adverse events is heightened, although many integrated interferences have been implemented (17–19). Therefore, the importance of reducing the recurrent adverse events in these patients is undisputed. Other potential risk factors or biomarkers may help to better identify these high-risk individuals, and further take targeted treatment measures to improve prognosis in this population.

In clinical practice, a considerable number of CAD patients are complicated with thyroid dysfunction. The relationship between thyroid disease and worse cardiovascular outcomes has been well studied (2, 3). Generally, overt and subclinical thyroid disorders increase the risk of adverse events in patients with CAD (6, 20). Moreover, variations in thyroid hormones, such as TSH, FT3 and FT4, have also been reported to be associated with CAD risk, without even exceeding the reference range (9, 21–24). TSH levels in the upper part of the reference range are suggested as a plausible marker of early-stage of hypothyroidism based on the fact that mild changes in serum thyroid hormone levels can lead to sensitive response of pituitary TSH secretion (25, 26). Some observational studies seemed to support this hypothesis and demonstrated that TSH within the high-normal range was significantly associated with CAD risk and mortality (24, 27, 28). In contrast, data from similar studies did not reveal significant association between high TSH levels and the risk of CAD events in euthyroid population (24). Moreover, other studies reported that high TSH levels might be beneficial and associated with lower risk of all-cause mortality in the oldest old (29). Inconsistent evidences were also found in the association between FT4 or FT3 levels and CAD risk in euthyroid patients (9, 10, 22). Taken together, these conflicting data reflects that we might be too obsessed with a certain type of thyroid hormone and ignored the dynamic variation of thyroid hormone metabolism.

Recently substantial research has reported that FT3/FT4 ratio, a surrogate marker evaluating degree of peripheral thyroxin deiodination and deiodinase activity, might stand for mild metabolic change of thyroid hormones relative exactly and is associated with unfavorable prognosis in some clinical settings. A study including 111 patients with dilated cardiomyopathy demonstrated low FT3/FT4 ratio was an indirect index of severe impairment in cardiac function and strongly correlated with poor prognostic markers like reduced LVEF as well as a high risk of subsequent mortality (11). Another prospective study reported FT3/FT4 ratio imbalance was significantly associated with frailty and increased mortality risk in older euthyroid patients hospitalized for acute disease (8). In the CAD setting, FT3/FT4 ratio was found to be related with long-term risk of all-cause and cardiac-related mortality in ACS patients (30). Similarly, an observational study showed FT3/FT4 ratio was a prognostic marker of all-cause death in euthyroid patients with AMI treated with PCI (10). In a previous publication, we also reported that FT3/FT4 ratio reduction increased risk of long-term cardiac death and MACCE in euthyroid patients with 3VD (12). However, it remains unclear whether FT3/FT4 ratio has predictive value in adverse events among euthyroid patients with established CVEs after PCI.

The result of the present study suggested that FT3/FT4 ratio was an independent predictor of long-term all-cause death, cardiac death and MACCE in this specific population, which was consistent with the findings of prior similar studies. This study was the first to identify FT3/FT4 ratio might be a potential marker of increased risk of adverse events in euthyroid patients with established CVEs treated with PCI. Peripheral thyroxin deiodination reflected by FT3/FT4 ratio is the main origin for the production of circulating T3, which is a deiodinated form of T4 and exert potent biological effect on targeted organ and tissues (31). During this process, deiodinase plays a crucial role. The down-regulation of deiodinase activity induces disturbance of thyroxin deiodination and reduction of FT3/FT4 ratio, and this condition exists in a variety of acute and chronic diseases including low T3 syndrome. Interestingly, we did observe that SPINA-GD, a structure parameter of thyroid homeostasis reflecting the sum activity of peripheral deiodinases, was decreased significantly with the declination of FT3/FT4 ratio. The underlying pathophysiological mechanism about the relationship between dysfunction of peripheral thyroxin deiodination and increased risk of adverse prognosis in CAD patients remains unclear. The reduction of peripheral thyroxin conversion is regarded as the general reaction to tissue injury and may indicate the severity degree of illness (32). In AMI patients, FT3/FT4 ratio was decreased and positively associated with amount of damaged heart tissue while the production rT3 was increased (32). Another plausible interpretation is that reduced thyroxin deiodination is related to antioxidant imbalance and inflammation, which involve in the atherosclerotic process (33). We did find that the CRP level, an established biomarker of inflammation status, was significantly higher in the group of lowest FT3/FT4 ratio. In addition, the decrease in conversion of T4 to T3 have a significant positive correlation with insulin resistance and abnormal glucose metabolism (34, 35). Despite these reasonable hypotheses, more studies are warranted to further investigate and confirm the exact mechanisms.

Clinical practice Guidelines on management of heart failure recommended routine assessment of thyroid function to detect overt or subclinical thyroid disorders (36). However, for euthyroid patients with prior CVEs and undergoing PCI, our study suggested that the mild alteration in peripheral thyroxin deiodination should also not be ignored. Routine evaluation of FT3/FT4 ratio might be a cheap and simple method to provide valuable clinical information. Whether there are targeted intervention strategies that can reduce the recurrent risk and improve the prognosis in this specific patient population need to be further investigated. Due to the slight variation of thyroid hormone levels among individuals with different sex and age, subgroup analysis was performed to investigate the potential interaction between FT3/FT4 ratio and sex, age and other common covariates. The result turned out to be consistent across different subgroups.

The current study has several limitations. First, thyroid function test was only performed at baseline, the thyroid function change during follow-up was not available. Second, the TPO antibody and rT3 level was not routinely tested and thyroid ultrasound was not routinely performed in our center, which might impair the precise evaluation of future risk of adverse events in CAD patients. Third, although we have adjusted as much necessary variates as possible, potential confounding may still exist.

## Conclusions

In euthyroid patients with prior cardiovascular events undergoing PCI, FT3/FT4 ratio might be a potential predictor of all-cause and cardiac mortality. Routine assessment of FT3/FT4 ratio might be a simple and effective tool for risk stratification in this specific patient population.

## Data Availability Statement 

The datasets presented in this article are not readily available due to ethical restrictions related to the consent given by subjects at the time of study commencement, our datasets are available from the corresponding author upon reasonable request after permission of the Institutional Review Board of Fuwai Hospital. Requests to access the datasets should be directed to dr_jinqingyuan@sina.com.

## Ethics Statement

The studies involving human participants were reviewed and approved by the Institutional Ethic Committee of Fu Wai Hospital. The patients/participants provided their written informed consent to participate in this study.

## Author Contributions

DY, XZ, RG, YY, BX, ZG and JY contributed to the conception and design of the work. SJ, PZ, CZ, YL, RL, JX and XT contributed to in data collection and analysis. DY drafted the manuscript. JY critically revised the manuscript. All authors contributed to the article and approved the submitted version.

## Funding

This work was supported by National Key Research and Development Program of China (No. 2016YFC1301300, 2016YFC1301301) and National Natural Science Foundation of China (No. 81770365).

## Conflict of Interest

The authors declare that the research was conducted in the absence of any commercial or financial relationships that could be construed as a potential conflict of interest.
