# Supplementary material for: Usefulness of FT3 to FT4 Ratio to Predict Mortality in Euthyroid Patients With Prior Cardiovascular Events Undergoing PCI: Five-Year Findings From a Large Single-Center Cohort Study
Source: Front Endocrinol (Lausanne). 2021 Jul 5;12:700349. doi: 10.3389/fendo.2021.700349 (PMC8287966; doi:10.3389/fendo.2021.700349)
Supplement: Supplementary file 1 [file Table_1.docx]

**Table S1. Univariable and multivariable analysis of the association between FT3/FT4 ratio and endpoints at 2 years.**

| **Outcomes** | **Crude HR (95% CI)** | **Crude P-value** | **Adjusted HR (95% CI)** | **Adjusted P-value** |
| --- | --- | --- | --- | --- |
| **All-cause death** |  |  |  |  |
| **T1** | 3.44 (1.56**‒7.58**) | **0.002** | 2.62 (1.11**‒6.21)** | **0.028** |
| **T2** | 1.98 (0.85**‒4.62)** | 0.116 | 1.92 (0**.**81**‒4.57)** | 0.140 |
| **T3** | Reference | ‒ | Reference | ‒ |
| **Cardiac death** |  |  |  |  |
| **T1** | 4.84 (1.65‒14.23) | **0.004** | 3.34 (1.04‒10.69) | **0.042** |
| **T2** | 1.98 (0.60‒6.56) | 0.266 | 1.82 (0.54‒6.20) | 0.335 |
| **T3** | Reference | ‒ | Reference | ‒ |
| **MACCE** |  |  |  |  |
| **T1** | 1.31 (1.05‒1.64) | **0.016** | 1.27 (0.99‒1.63) | 0.064 |
| **T2** | 0.87 (0.69‒1.11) | 0.275 | 0.85 (0.66‒1.09) | 0.203 |
| **T3** | Reference | ‒ | Reference | ‒ |
| **MI** |  |  |  |  |
| **T1** | 1.01 (0.60‒1.73) | 0.958 | 0.99 (0.54‒1.82) | 0.971 |
| **T2** | 0.76 (0.43‒1.36) | 0.361 | 0.76 (0.42‒1.36) | 0.350 |
| **T3** | Reference | ‒ | Reference | ‒ |
| **Stroke** |  |  |  |  |
| **T1** | 0.75 (0.37‒1.55) | 0.444 | 1.06 (0.51‒2.20) | 0.885 |
| **T2** | 1.26 (0.67‒2.39) | 0.477 | 0.73 (0.35‒1.54) | 0.409 |
| **T3** | Reference | ‒ | Reference | ‒ |
| **Revascularization** |  |  |  |  |
| **T1** | 1.18 (0.91‒1.54) | 0.214 | 1.25 (0.92‒1.68) | 0.149 |
| **T2** | 0.85 (0.64‒1.13) | 0.262 | 0.84 (0.63‒1.13) | 0.248 |
| **T3** | Reference | ‒ | Reference | ‒ |

Model adjusted for age, sex, BMI, diabetes, hypertension, dyslipidemia, family history of CAD, smoking, clinical presentation (stable angina pectoris or ACS), LVEF, TSH, T3, T4, HbA1c, LDL-C, hs-CRP, eGFR, lesion vessels, left main/three vessel disease, SYNTAX score, complete revascularization, number of stents, DES implantation.

CI, confidence interval; HR, hazard ratio; MACCE, major adverse cardiac and cerebrovascular events; MI, myocardial infarction.

**Table S2. Subgroup analysis on MACCE across tertiles of FT3/FT4 ratio.**

|  | **No. of Patients with events (%)** | | | **Adjusted HR (95% CI)** | | | **P for interaction** |
| --- | --- | --- | --- | --- | --- | --- | --- |
| **FT3/FT4 ratio** | **T1** | **T2** | **T3** | **T1 vs T3** | **T2 vs T3** | **T1vs T2** |  |
| **Age** |  |  |  |  |  |  | 0.516 |
| **＜65 years** | 230 (28.9) | 186 (21.1) | 212 (22.5) | 1.37 (1.11‒1.69) | 0.94 (0.77‒1.15) | 1.46 (1.20‒1.79) |  |
| **≥65years** | 102 (27.3) | 72 (22.8) | 53 (22.2) | 1.26 (0.87‒1.82) | 1.04 (0.72‒1.50) | 1.21 (0.88‒1.67) |  |
| **Sex** |  |  |  |  |  |  | 0.506 |
| **Male** | 261(29.8) | 221(22.0) | 235(22.4) | 1.41(1.15-1.71) | 0.99(0.82-1.20) | 1.42(1.18-1.71) |  |
| **Female** | 71(24.1) | 37(19.3) | 30(22.7) | 0.90(0.56-1.45) | 0.67(0.41-1.10) | 1.34(0.88-2.05) |  |
| **Diabetes** |  |  |  |  |  |  | 0.161 |
| **Yes** | 188(27.9) | 130(22.7) | 128(25.5) | 1.14(0.89-1.47) | 0.88(0.69-1.13) | 1.30(1.03-1.64) |  |
| **No** | 144(29.0) | 128(20.4) | 137(20.1) | 1.51(1.17-1.96) | 1.01(0.79-1.30) | 1.49(1.17-1.91) |  |
| **Clinical presentation** |  |  |  |  |  |  | 0.407 |
| **SAP** | 147(27.5) | 121(20.0) | 152(22.9) | 1.23(0.95-1.59) | 0.84(0.66-1.08) | 1.46(1.14-1.88) |  |
| **ACS** | 185(29.1) | 137(23.1) | 113(21.9) | 1.50(1.16-2.00) | 1.10(0.85-1.42) | 1.37(1.08-1.73) |  |
| **Left Main/three-vessel Disease** |  |  |  |  |  |  | 0.660 |
| **Yes** | 29(32.2) | 26(32.9) | 23(24.2) | 1.11(0.92-1.35) | 1.23(0.96-1.59) | 1.26(1.01-1.59) |  |
| **No** | 303(28.1) | 232(20.7) | 242(22.3) | 1.40(1.08-1.81) | 0.93(0.71-1.21) | 1.51(1.17-1.94) |  |
| **SYNTAX score** |  |  |  |  |  |  | 0.374 |
| **0-22** | 291(28.1) | 227(20.7) | 232(21.6) | 1.38(1.14-1.68) | 0.96(0.80-1.16) | 1.44(1.20-1.72) |  |
| **23-32** | 32(30.2) | 24(27.3) | 26(28.9) | 0.94(0.52-1.70) | 0.86(0.47-1.58) | 1.09(0.61-1.93) |  |
| **≥33** | 9(32.1) | 7(43.8) | 7(43.8) | 0.60(0.14-2.62) | 0.81(0.17-3.82) | 0.74(0.18-2.98) |  |
